# Sex-specific estimation of *cis* and *trans* regulation of gene expression in heads and gonads of *Drosophila melanogaster*

**DOI:** 10.1093/g3journal/jkad121

**Published:** 2023-06-01

**Authors:** Gemma Puixeu, Ariana Macon, Beatriz Vicoso

**Affiliations:** Institute of Science and Technology Austria, Am Campus 1, Klosterneuburg 3400, Austria; Institute of Science and Technology Austria, Am Campus 1, Klosterneuburg 3400, Austria; Institute of Science and Technology Austria, Am Campus 1, Klosterneuburg 3400, Austria

**Keywords:** *cis*-regulatory variation, *trans*-regulatory variation, sex-biased gene expression, testis, additivity of gene expression

## Abstract

The regulatory architecture of gene expression is known to differ substantially between sexes in *Drosophila*, but most studies performed so far used whole-body data and only single crosses, which may have limited their scope to detect patterns that are robust across tissues and biological replicates. Here, we use allele-specific gene expression of parental and reciprocal hybrid crosses between 6 *Drosophila melanogaster* inbred lines to quantify *cis*- and *trans*-regulatory variation in heads and gonads of both sexes separately across 3 replicate crosses. Our results suggest that female and male heads, as well as ovaries, have a similar regulatory architecture. On the other hand, testes display more and substantially different *cis*-regulatory effects, suggesting that sex differences in the regulatory architecture that have been previously observed may largely derive from testis-specific effects. We also examine the difference in *cis*-regulatory variation of genes across different levels of sex bias in gonads and heads. Consistent with the idea that intersex correlations constrain expression and can lead to sexual antagonism, we find more *cis* variation in unbiased and moderately biased genes in heads. In ovaries, reduced *cis* variation is observed for male-biased genes, suggesting that *cis* variants acting on these genes in males do not lead to changes in ovary expression. Finally, we examine the dominance patterns of gene expression and find that sex- and tissue-specific patterns of inheritance as well as *trans*-regulatory variation are highly variable across biological crosses, although these were performed in highly controlled experimental conditions. This highlights the importance of using various genetic backgrounds to infer generalizable patterns.

## Introduction

Variation in gene expression has been shown to underlie human disease and contribute to trait evolution between closely related species, and understanding mutational and selective processes driving it has been a key goal in evolutionary biology (Emerson and Li 2010; Signor and Nuzhdin 2018). Genetic variants that contribute to the inheritable component of this variation can modulate gene expression either in *cis* or in *trans*. *Cis* variants only affect the expression of a linked allele (e.g. mutations at a gene promoter or enhancer), whereas *trans*-acting variants can affect the expression of both copies of close or distant genes (e.g. mutations that change the activity or expression of a transcription factor, reviewed in Signor and Nuzhdin (2018)).

Two main approaches have been employed for studying the evolution of gene regulation within and between species and the contribution of *cis* and *trans* variants. Expression quantitative trait loci (eQTLs) can uncover the local and distal regulatory architecture of gene expression variation, which are typically assigned as acting in *cis* and *trans* based on a distance cutoff (Bhasin *et al*. 2008; Massouras *et al*. 2012). A more mechanistic assessment of *cis* and *trans* effects has come from comparisons of parental lines or species and allele-specific expression in heterozygous hybrid crosses, as *trans* regulators should modulate the expression of both gene copies in the hybrid, whereas *cis* regulators lead to allelic imbalances in the hybrid (Wittkopp *et al*. 2004; Graze *et al*. 2009). While unable to pinpoint specific genetic variants underlying the regulation, these hybrid studies provide an estimate of the total *cis* and *trans* regulation affecting individual genes, with both types of regulation being common (Hughes *et al*. 2006; Wittkopp *et al*. 2008; Metzger *et al*. 2016). This approach also provides information on the level of dominance of regulatory variants and has shown that *cis*-acting variants are typically closer to additivity than *trans*-acting variants (Lemos *et al*. 2008; McManus *et al*. 2010; Meiklejohn *et al*. 2014).

Comparisons of estimates of *cis* and *trans* effects over various distances between parental lines show that while *trans* variants control most of the variation within species, *cis* variants appear to disproportionately contribute to differences between species (Wittkopp *et al*. 2008; Coolon *et al*. 2014; Metzger *et al*. 2017), either because they are less pleiotropic or because their increased additivity (Lemos *et al*. 2008; McManus *et al*. 2010; Zhang *et al*. 2011; Gruber *et al*. 2012; Meiklejohn *et al*. 2014) and larger effect sizes (Brem *et al*. 2002; Schadt *et al*. 2003; Hughes *et al*. 2006; Gruber *et al*. 2012; Metzger *et al*. 2016) give them a selective advantage over *trans* variants. Importantly, the regulatory architecture and contribution of *cis* and *trans* variants vary depending on the population under study (Martin *et al*. 2014), the sampled tissue (GTEx Consortium *et al*. 2017; Glaser-Schmitt *et al*. 2018; Benowitz *et al*. 2020) and sex (Meiklejohn *et al*. 2014; Oliva *et al*. 2020), and the environment (Chen *et al*. 2015; Fear *et al*. 2016; Buchberger *et al*. 2019), and this has potentially important consequences for how selection acts on genes expressed in these different contexts (Chen *et al*. 2015; Buchberger *et al*. 2019).

Genes that are expressed exclusively or preferably in one sex have been of particular interest, as they show unusual patterns of divergence of gene expression (Ellegren and Parsch 2007). Genes that are primarily expressed in the testis often evolve unusually quickly in arthropods at both the sequence and expression levels (Meiklejohn *et al*. 2003; Whittle *et al*. 2021). This is thought to be due to sexual selection (Ellegren and Parsch 2007), as well as to the low pleiotropy of many genes expressed in the testis (Meisel 2011). Ovary-biased genes, on the other hand, tend to show either no or small increases in divergence rates compared with unbiased genes (Ellegren and Parsch 2007; Sackton *et al*. 2014; Whittle and Extavour 2019; Whittle *et al*. 2021; but see Whittle and Extavour (2017) for an exception in mosquitoes). In the soma, the relationship between sex-biased expression and rates of evolution has been mostly studied in heads and brain tissues (Khodursky *et al*. 2020; Whittle *et al*. 2021). In that case, both female-biased and male-biased genes appear to have faster expression divergence than unbiased genes (Khodursky *et al*. 2020; Whittle *et al*. 2021). Consistent with these unusual evolutionary patterns, gene regulation also appears to vary between females and males. In *Drosophila*, variants with a sexually dimorphic effect on expression often act in *cis* (Meiklejohn *et al*. 2014), and genes with female-biased expression carry more *cis* variants (Mishra *et al*. 2022). The dominance of regulatory variants can also differ between sexes, with deviations from the additivity of *cis* variants acting in opposite directions in the two sexes in *Drosophila* hybrids (Meiklejohn *et al*. 2014) and with males generally showing more additive effects than females in nematode hybrids (Sánchez-Ramírez *et al*. 2021). Despite this long-standing interest in the regulation and evolution of sex-biased genes, direct comparisons of estimates of *cis* and *trans* effects in gonads and somatic tissues are rare. It is therefore unclear if these differences relate to the germline-specific regulatory architecture or to more general differences in how males and females control gene expression. Mishra *et al*. (2022) recently found that sex-specific *cis* effects were more common in the gonad, but total *cis* effects were not.

The prevalence of *cis* and *trans* effects found in different categories of genes also has the potential to shed some light on what selective pressures are acting on gene expression (Emerson *et al*. 2010; Coolon *et al*. 2014). For instance, genes known to be under strong selective constraints show less *cis*- and *trans*-driven expression variation than other genes, consistent with stabilizing selection on gene expression (Emerson *et al*. 2010). Mishra *et al*. (2022) recently predicted an excess of *cis* effects for genes for which gene expression is evolving under sexual antagonism, as mutations that increase or decrease expression may be under balancing selection. Contrary to their expectations, genes with intermediate levels of sex bias (SB), which are thought to be more often under sexual conflict (Cheng and Kirkpatrick 2016), did not harbor an excess of *cis* variants. Instead, the prevalence of *cis* effects increased with increasing female bias, but why this occurred was unclear. More generally, both positive selection and negative selection should decrease the amount of polymorphic *cis* and *trans* variants within a population, while balancing selection should lead to their maintenance.

Here, we systematically estimate *cis* and *trans* effects, as well as dominance, acting on gene expression in the soma and germline of *Drosophila melanogaster*. We performed pairwise crosses between 6 inbred lines of the *Drosophila* Genetic Reference Panel (DGRP) (Mackay *et al*. 2012; Huang *et al*. 2014), such that we had three hybrid crosses as independent biological replicates, and obtained RNA-seq reads for both heads and gonads of females and males. Our results highlight the unusual regulatory architecture of the testis and suggest that the expression of genes that are sex-biased is under different levels of *cis* regulation compared with unbiased genes. Finally, despite the highly correlated patterns of expression across samples in our dataset, results varied substantially between crosses, highlighting the limitations of studying these patterns in a single genetic context.

## Materials and methods

### Sample preparation and sequencing

We obtained the sex-specific replicated gene expression for heads and gonads from crosses within and between *D. melanogaster* inbred lines. Specifically, we randomly selected six lines from the DGRP (*Drosophila* Genetic Reference Panel, Mackay *et al*. 2012; Huang *et al*. 2014) without *Wolbachia* infection and main inversions (Huang *et al*. 2014) and matched them into 3 pairs: DGRP-757 × DGRP-392, DGRP-208 × DGRP-808, and DGRP-83 × DGRP-332. Crosses within and between lines were set up in vials containing 40 males and 40 virgin females (between 1 and 5 vials depending on the number of individuals that could be obtained), at 23°C under a 12-h light/12-h dark cycle. For each cross, we then dissected 2 replicate samples of heads and gonads of 20 4-day-old virgin females and virgin males for each within-line cross and reciprocal between-line cross, obtaining a total of 96 samples (experimental design outlined in Supplementary Fig. 1). Both replicates contained individuals pooled across vials to avoid biases due to variation across microenvironments.

Samples were flash frozen in liquid nitrogen and kept at −80°C until further processing. RNA was then extracted with a Maxwell RSC simplyRNA Tissue Kit (Promega). Two Smart-seq2 RNA-seq libraries were produced from tagged and pooled samples at the Vienna Biocenter Sequencing Facility (1 for each replicate) and sequenced on an Illumina NovaSeq machine (single-end 100-bp reads).

### Data processing and (allele-specific) expression estimation

We obtained demultiplexed data for each of the 2 libraries, each containing 48 samples and corresponding respectively to replicates R1 and R2 of each tissue/sex. We trimmed the data using Trimmomatic (Bolger *et al*. 2014) and performed UMI-based deduplication using UMI-tools (Smith *et al*. 2017). The final dataset consisted of 5.7 to 10.6 million reads per sample, except for sample 808M × 208F male testes R2, which had only about 15,000 reads and was removed from the analysis. We estimated overall count and TPM (transcripts per million) gene expression using Kallisto (Bray *et al*. 2016), reported in Supplementary Datasets 1 and 2, respectively. Two further samples were removed because of their low correlation to other samples of the same tissue: 392M × 392F male testes R2 and 392F × 757M male heads R2 (Spearman’s correlation < 0.8). All subsequent analyses were done without these 3 samples.

To estimate allele-specific expression, we followed the pipeline described in Takada *et al*. (2017). In short, we reconstructed the genotypes of the 6 parental lines using VCFtools (Danecek *et al*. 2011), from a VCF file containing information on all the DGRP lines, and the corresponding dm3 reference genome sequence, eliminating indels and only keeping SNPs. We then estimated allele-specific expression by mapping RNA-seq reads to transcriptomes reconstructed from parental genomes. The line-specific transcriptomes were generated from the reconstructed genotypes using the Ensembl GTF file (version dm3, obtained from https://hgdownload.soe.ucsc.edu). We mapped the RNA-seq on the transcriptomes using Bowtie 2 (Langmead and Salzberg 2012) and estimated allele-specific expression using ASE-Tigar (Nariai *et al*. 2016). Because allele-specific expression data cannot be accurately estimated for genes with minimal variation between parental lines, we only used transcripts with at least 3 exonic single nucleotide variants between parental lines. We then summed FPKM (fragments per kilobase of transcript per million mapped reads) and estimated counts per transcript across isoforms to obtain expression levels per gene. To avoid biases in males and for consistency in females, X-linked genes were removed, so only autosomal data were used for all the subsequent analyses.

This same pipeline was used to estimate overall parental expression between pairs of parental lines from a file containing the reads of both parentals pooled together so that the hybrid allelic and overall parental expression estimates are comparable to 1 another. Hybrid allelic expression and overall parental expression are in Supplementary Datasets 3 and 4 as count and FPKM estimates, respectively. Hybrid allelic expression data were used to estimate *cis*-regulatory (CR), parent-of-origin (PO), and maternal genotype (MG) effects via the Takada *et al*. (2017) pipeline, and both hybrid allelic expression and overall parental expression were used to estimate *cis*- and *trans*-regulatory effects via the McManus *et al*. (2010) pipeline.

### Estimation of CR, PO, and MG effects

We adapted a pipeline developed by Takada *et al*. (2017) to estimate CR, PO, and MG effects by modeling the allele-specific gene expression as count data as a function of the 3 binary fixed effects: *E* ∼ *µ* + CR + PO + MG + *ε*. We defined separate models per sex, tissue, and cross, leading to a total of 12 models, each (except for those with some missing samples; see above) including 8 data points: expression for alleles A and B in 2 replicates of each reciprocal cross. For CR, we assigned 0 (1) for A (B); for PO, 0 (1) was assigned if the chromosome was inherited from the mother (father); and for MG, 0 (1) was assigned to samples from cross A × B (B × A) (see Takada *et al*. (2017) for more details on the models). We included in the analyses all genes with TPM > 1 in at least 4 of the 8 samples. We defined negative binomial generalized linear models (GLMs), modeling the RNA-seq as count data using the edgeR library (McCarthy *et al*. 2012) in the R statistical package (R Core Team 2022). Significance was determined at Benjamini-Hochberg false discovery rate (FDR)-corrected *P*-values of <0.05. We also computed the deviance explained by CR as the difference in the total deviance in allele-specific expression explained by the full model without CR (only with PO and MG) and the full model including CR. The data used for this analysis can be found in Supplementary Dataset 3.

### Sex- and tissue-dependent CR effects

We extended the models above by including tissue-specific (sex-specific) samples of both sexes (tissues) to examine how CR interacts with sex (tissue). Sex differences in CR effects can be due to differences in magnitude or direction, the first being variation that affects gene expression more strongly in 1 sex than the other and the second being mutations that lead to increases in gene expression in 1 sex and decreases in the other (i.e. sex reversal). To be able to disentangle between the 2, we defined 2 types of models. First, we modeled allele-specific expression as *E* ∼ *µ* + CR + sex + CR*sex + *ε*. Second, we modeled allele-specific expression as *E* ∼ *µ* + CRsex + *ε*, CRsex being CR recoded to explicitly be contrary in the 2 sexes: A (B) being 0 (1) in females and 1 (0) in males. While the first strategy captures significant interactions between CR and sex, which would include both sex differences of different magnitude and direction, the second only looks for differences in direction, explicitly giving an idea of the extent of sex reversal in CR.

The exact same strategy was applied to examine tissue differences in CR.

### Extent of CR effects across SB levels

To examine whether the extent of CR effects differs with SB, we compared the deviance in allele-specific gene expression explained by CR effects across genes (estimated using the negative binomial GLMs) belonging to different SB categories. SB was determined as SB = log_2_[(exp_F_ + 1)/(exp_M_ + 1)] for each cross and tissue separately. Genes were split into 5 SB categories: strongly male-biased (MS), SB < −1; male-biased (MB), −1 < SB < −0.3; unbiased (UB), −0.3 < SB < 0.3; female-biased (FB), 0.3 < SB < 1; and strongly female-biased (FS), SB > 1. Within-sex statistical comparisons of deviances explained across SB categories were done with the Mann–Whitney *U* test in Python.

### Overlap of CR effects between samples

We next examined whether the genes showing CR are the same across samples by testing the significance in the overlap between CR hits across all pairs of samples. Concretely, we defined contingency tables by determining which genes have equal CR categorization in the 2 samples considered: CR in both (shared CR), CR in 1 or the other, and non-CR in both, at FDR < 0.05. Next, we applied *χ*^2^ tests to determine the significance of the under- or overrepresentation of the shared CR category.

### *Cis*- and *trans*-regulatory divergence assignment

We classified genes into various *cis*- and *trans*-regulatory categories by following the pipeline described in McManus *et al*. (2010). For this, we used allele-specific count expression data in the hybrids and overall expression in the parentals, both of which were estimated using the ASE-Tigar pipeline described above. We made the classification separately for each sex, tissue, and cross, pooling the reciprocals together (so that for each parental and hybrid, we have a total of 2 and 4 samples, respectively, except for those crosses with missing data) and only using genes where the sum of estimated reads in the 2 parental lines was at least 20. The data used for this analysis can be found in Supplementary Dataset 3.

We determined whether there was a significant difference in expression between parentals (*P*), between the two alleles in hybrids (*H*) and a *trans* (*T*) effect for each particular gene. *P* and *H* effects were determined via statistical tests (DEseq2, Love *et al*. 2014). *P* expression was considered differential if the FDR for differential expression between the 2 parentals was less than 0.05. The same threshold was used to determine differential expression between the 2 alleles in *H* samples. *T* effects were determined by comparing the allele-specific mRNA abundance difference between the *P* and *H* samples using Fisher's exact test followed by FDR analysis and considered significant at FDR < 0.05. Using a custom Python script, we classified genes into the following 7 categories by comparing the significance classifications from all the 3 tests:

1. Conserved: no significant differential expression in *P* or *H*. No significant *T*.
2. *Cis* only: significant differential expression in *P* and *H*. No significant *T*.
3. *Trans* only: significant differential expression in *P* but not in *H*. Significant *T*.
4. *Cis* + *trans*: significant differential expression in *P* and *H*. Significant *T*. log_2_(P1/P2)/log_2_(A1/A2) > 1. *Cis*- and *trans*-regulatory effects favor the expression of the same allele.
5. *Cis* × *trans*: significant differential expression in *P* and *H*. Significant *T*. log_2_(P1/P2)/log_2_(A1/A2) < 1. *Cis*- and *trans*-regulatory effects favor the expression of the different alleles.
6. Compensatory: significant differential expression in *H* but not in *P*. Significant *T*. Expression differences caused by *cis*- and *trans*-regulatory components have an opposite direction and perfectly compensate each other such that there is no expression difference in *P*.
7. Ambiguous: significant in only 1 of differential expression tests in *P*, *H*, or *T*. Thus, no explicit *cis*/*trans* effect can be detected.

### Inheritance pattern classification

The mode of inheritance was determined for genes that are differentially expressed across the two parental lines (with a fold difference between the two parents of at least 1.5) and where the sum of estimated reads in the two parental lines was at least 20, separately for each sex, tissue, and cross, by averaging across reciprocals.

We adapted the pipeline developed by Gibson *et al*. (2004) to classify the genes into the various inheritance categories using a 1.25-fold change TPM expression cutoff between overall expression estimated by Kallisto (ignoring allele-specific expression) in parentals vs hybrids. We considered that genes whose expression in hybrids deviated from that of either parent have nonconserved inheritance and classified them into the following categories: additive genes are those where hybrid expression was 1.25-fold greater than one parent and less than the other; overdominant (underdominant) genes were 1.25-fold greater (less) than both parents; and dominant genes were only different from one of the two parents. The data used for this analysis can be found in Supplementary Dataset 2.

## Results

We randomly chose six *D. melanogaster* inbred lines from the DGRP (Mackay *et al*. 2012, Huang *et al*. 2014) without *Wolbachia* infection and major inversions (Huang *et al*. 2014) and matched them pairwise: DGRP-757 × DGRP-392, DGRP-208 × DGRP-808, and DGRP-83 × DGRP-332. For each pair, we performed within-line crosses as well as the 2 between-line reciprocals and obtained 2 replicated measures of sex-specific gene expression in heads and gonads for each of the 4 crosses per pair (see Supplementary Fig. 1 for a schematic representation of the experimental design).

### More *cis* variation in testes than in ovaries and heads

**Fig. 1.**
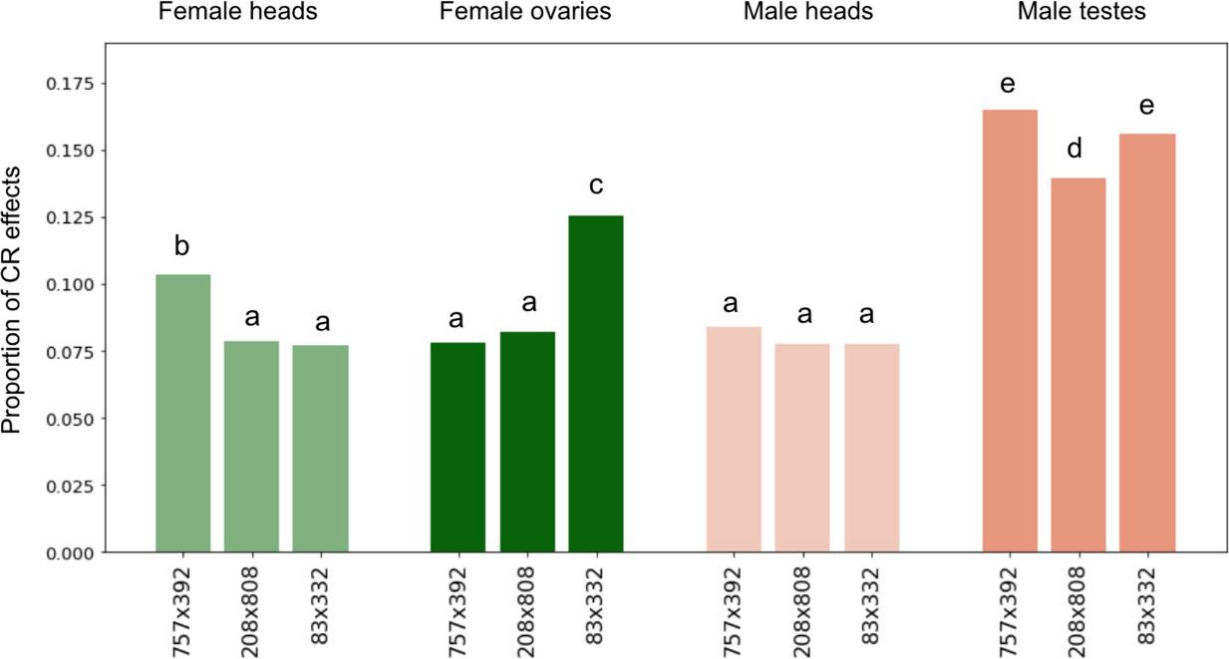
Sex-, tissue-, and cross-specific proportion of CR effects. CR effects have been determined by modeling allele-specific expression (*E*) as a function of CR, PO, and MG effects as *E* ∼ *µ* + CR + PO + MG + *ε*, as described in *M*aterials and m*ethods*. Significance groups revealing differences between proportions of genes with significant CR effects across all samples (Fisher's exact test at *P*-value < 0.05) are denoted by different letters (a–e).

We took two complementary approaches to evaluate the extent of CR variation in female and male heads and gonads. First, we implemented the GLM of Takada *et al*. (2017), which models replicated allele-specific expression data in reciprocal crosses (*E*) as a function of CR effects on expression. This method also considers potential PO effects (e.g. imprinting) and MG effects (e.g. due to mitochondria or maternal RNAs deposited in the egg), which are thought to be rare in *Drosophila* (Wittkopp *et al*. 2006; Coolon *et al*. 2012; Chen *et al*. 2015; Takada *et al*. 2017), but can potentially bias estimates of *cis* variation and so are included in the model as covariates: *E* ∼ *µ* + CR + PO + MG + *ε*, where *µ* and *ε* are the estimated average and error term. Since X chromosomes are hemizygous in males, we focus on autosomal genes. Supplementary Table 1 confirms the near absence of parental or MG effects. The detection of MG effects almost exclusively in males indicates that those might be X-downstream effects. In line with previous results, we found widespread CR effects, with 7.7–10.3, 7.7–8.4, 7.8–13.6, and 13.9–16.5% of genes showing significant *cis* effects in female heads, male heads, female ovaries, and male testes across replicate crosses. The proportion of genes that are *cis*-regulated is higher in the testis than in the other three tissues for all crosses (Fig. 1, *P* < 0.05 for all Fisher's exact comparisons), showing that differences between sexes are largely driven by the testis.

**Fig. 2.**
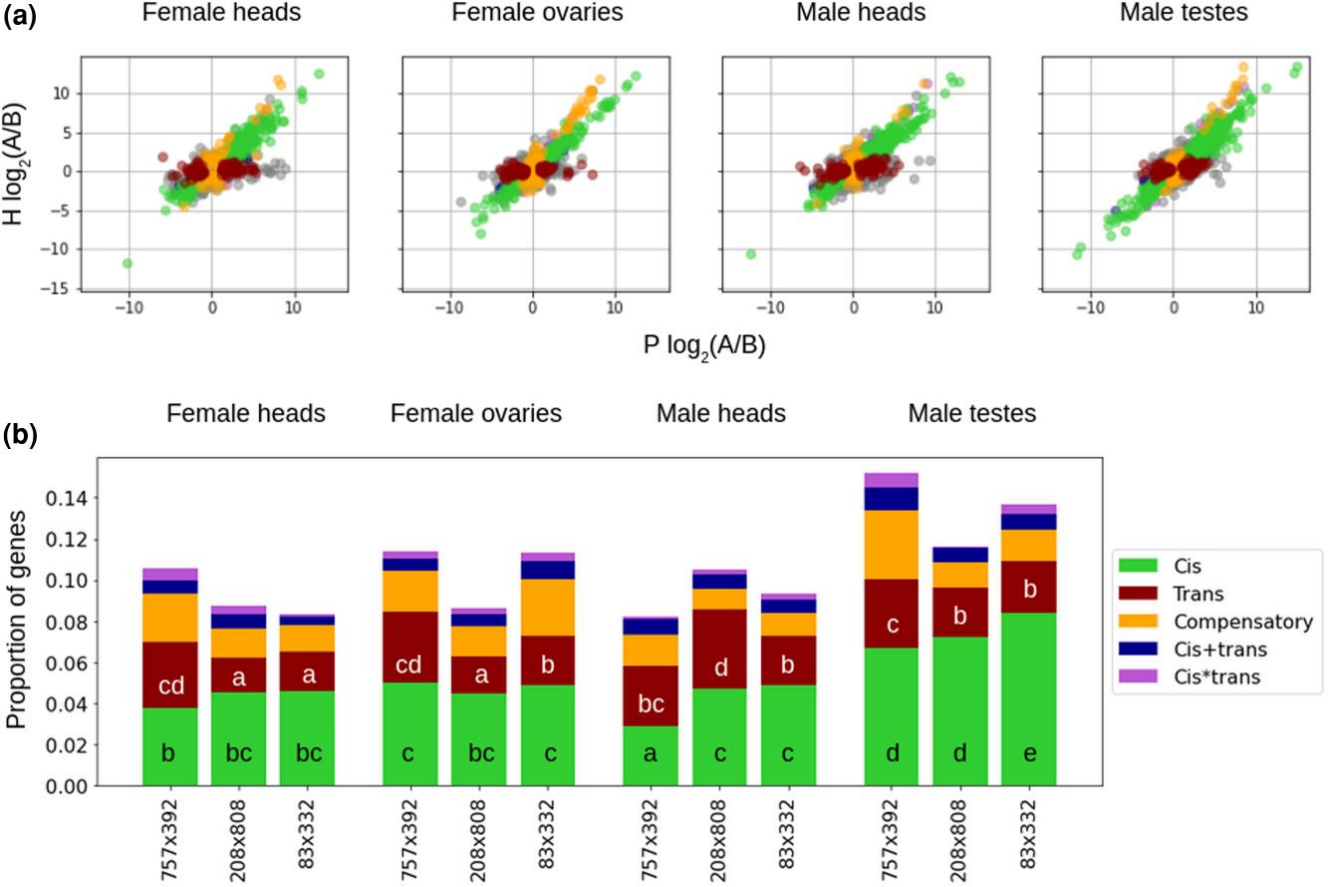
Patterns of *cis*- and *trans*-regulatory variation. a) Scatter plots of the relative allele-specific expression levels in parental (*P*) vs hybrid (*H*) (averaged across reciprocals) datasets in each sex and tissue for the cross 83 × 332 as a representative example (but see Supplementary Fig. 2 for all the plots). Each dot is a different gene and is color-coded according to the mechanism of expression regulation, inferred via hierarchical classifications based on significant expression differences in allelic expression between *P* and *H*. b) Proportion of genes displaying each of the expression regulation mechanisms in each sex, tissue, and cross. Significance groups revealing differences in proportions of genes displaying *cis* and *trans* regulation (in black and white, respectively) across all samples (2-proportions *z*-test at *P*-value < 0.05) are denoted by different letters (a–e and a–d).

The second approach follows the pipeline developed by McManus *et al*. (2010), which uses allele-specific expression estimates in the hybrids together with overall expression in both parental lines to estimate both *cis*- and *trans*-regulatory effects. Specifically, genes that have the same ratio of expression between the two parental alleles in the hybrids as between the parentals themselves are likely under *cis* regulation, while genes without allelic imbalances in the hybrids given differences in expression between the parentals are likely under *trans* regulation. We applied this approach to each of the tissues, sexes, and crosses, using differential expression tests (DEseq2) to call differences between allelic expression in the parents and hybrids (Fig. 2a; see *M*aterials and m*ethods*). We again found evidence of increased CR variation in testes (*P* < 0.05 for all comparisons, 2-proportions *z*-test), while the extent of *trans* regulation was highly variable across sexes, tissues, and crosses (Fig. 2b). We only had 2 replicates per sample, which limited the power to detect differentially expressed genes. Since we found little evidence of PO and MG effects in the previous analysis (Supplementary Table 1), which indicates that the reciprocal crosses behave largely as biological replicates, we used hybrids derived from reciprocal crosses as replicates for the main analysis. Importantly, we find that the main patterns hold when looking at reciprocals separately (Supplementary Fig. 3). The CR effects identified using both the McManus *et al*. (2010) and Takada *et al*. (2017) pipelines are also highly concordant, with *P*-values < 10*e*−16 for all comparisons between the 2 (*χ*^2^ on contingency tables as CR vs non-CR using the 2 methods).

The excess of *cis* variation in the testis could be due to testis-specific genes harboring more genetic variants or to genetic variants on broadly expressed genes causing more variation in gene expression in the testis. To investigate this, we inferred the extent of testis specificity of each gene as the proportion of its total expression in the FlyAtlas 2 database (Leader *et al*. 2018) that came from the testis. We then checked if the excess of *cis* effects (inferred using the GLM) was driven by testis-specific genes. A lack of correlation between testis specificity and CR FDR-corrected *P*-values (Spearman’s rank correlation of −0.002, 0.001, and −0.024 for each of the 3 crosses and corresponding *P*-values of 0.881, 0.974, and 0.053) suggests that the enrichment for *cis* effects in testes is at least partly a consequence of testis-specific regulation of broadly expressed genes rather than a property of testis-specific genes. It should be noted that other tissue-specific properties, such as differences in mean or variance in expression, may also contribute to the detection of an excess of CR variation in the testis.

Finally, we looked at the overlap of genes that are under CR effects across sexes, tissues, and crosses (Supplementary Fig. 4). We find a significant overlap of CR effects between heads of the 2 sexes and ovaries, indicating that the standing CR genetic variation is similarly used between these tissues. However, significantly fewer genes than expected have shared CR effects in the testis and the other tissues, which presents further evidence that this tissue has its own CR landscape.

### Extensive sex-specific CR effects in the gonads

We next investigated whether there are sex differences in CR effects, i.e. if the same genetic variants affect male and female expression differently. Such “sex-by-*cis* effects” are evidence of a sex-specific regulatory architecture and are thought to contribute to the decoupling of the genotype-to-phenotype relationship between sexes, allowing sex-specific (expression) traits to evolve independently toward their optima (Stewart *et al*. 2010). We detected sex differences in *cis* regulation by modeling the sex-by-CR effect interaction on gene expression: *E* ∼ *µ* + sex + CR + sex*CR + *ε*. We detected between 7.5 and 11.1% of genes across crosses having a significant sex-by-CR effect interaction in gonads, while only 0.3–1.1% were significant in heads, suggesting that the regulatory architecture is highly shared between sexes in heads but substantially sex-specific in gonads. This is consistent with sex differences in allelic usage being an important genetic mechanism contributing to sexual dimorphism.

Sex differences in CR effects can be of different magnitude or direction, the latter consisting of sex reversal in allele-specific expression. This extreme case of differential allelic imbalances between sexes might be maintained by sexually antagonistic balancing selection (Kidwell *et al*. 1977; Connallon and Chenoweth 2019). We detected sex reversal in allelic imbalance by explicitly modeling a scenario where allelic usage is opposite across sexes (see *Material*s *and methods*). We find that sex reversal in CR effects is rare in gonads (0.1–1%) and almost absent in heads (0.0–0.1%).

We used a similar strategy to detect tissue differences in CR effects. We modeled sex-specific tissue-by-*cis* effect interaction to detect tissue differences in allelic usage and opposite allele-specific usage across tissues to detect tissue reversal. We find a significant tissue-by-CR effect in 4.6–7.0% of genes in females and in 10.2–13.0% of genes in males across replicate crosses. The higher extent of tissue differences in allelic usage in males is further evidence of the testis-specific CR architecture.

### Sex-specific CR effects across SB categories

To test the hypothesis that genes of intermediate SB, likely under the strongest sexual conflict (Cheng and Kirkpatrick 2016), have more CR variation (Mishra *et al*. 2022), we determined, for each gene, how much of the deviance in their expression was explained by CR effects in our GLM in both tissues and sexes separately. We then compared the distribution of the deviation explained by *cis* effects across genes of different SB categories in heads and gonads separately.

**Fig. 3.**
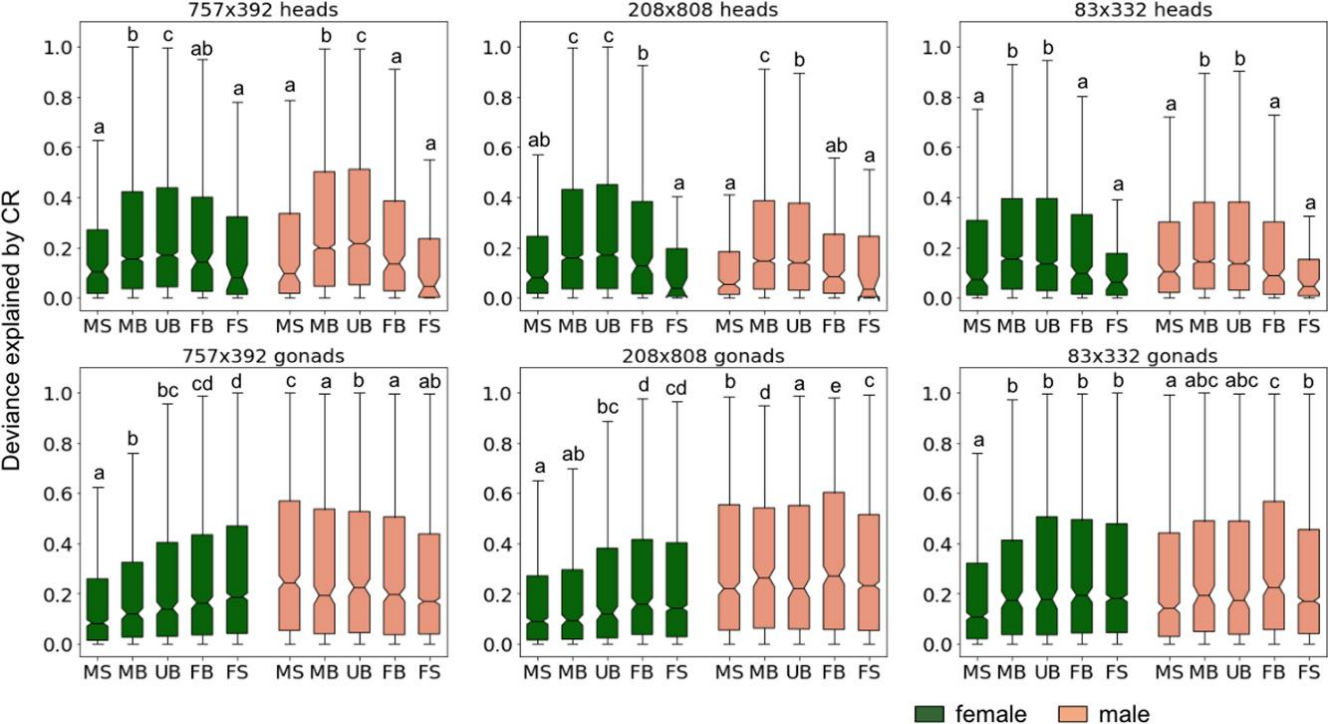
Deviance in allelic gene expression explained by CR effects in each SB category for each tissue and cross in females (green) and males (orange). Each gene was classified into 5 SB categories: MS (strongly male-biased), MB (male-biased), UB (unbiased), FB (female-biased), and FS (strongly female-biased; see *Methods* for specific SB thresholds). CR deviances were calculated for each sex as the deviance in allele-specific expression explained by CR while correcting for the other effects considered (see *M*aterials and m*ethods*). Significance groups revealing differences between SB groups within each cross, tissue, and sex (Mann–Whitney *U* tests at *P*-value < 0.05) are denoted by different letters (a–e).

Figure 3 shows that, in heads, the amount of CR deviation forms an inverted U-shape along categories of SB: FS and MS genes have very low CR variation in both female and male heads, while UB or moderately MB genes show the most variation. In gonads, the picture is less consistent across sexes and crosses. In ovaries, there is a decrease in CR variation in MS genes relative to UB and FB genes. In testes, we find a symmetric pattern to that of ovaries in 1 of the replicate crosses [less testis *cis* variation in (strongly) FB genes, although this is not significant]. In the other 2 crosses, we find the expected enrichment in *cis* variation for genes with intermediate levels of SB (Fig. 3), but this difference is only significant for 1 of the 3 crosses.

### Additive genes have more CR effects

Lastly, we tested whether there is a relationship between the molecular mechanisms of regulatory divergence and the degree of additivity of expression. We determined the degree of additivity of expression of each gene by comparing overall expression between parental and hybrid lines for each sex, tissue, and cross independently, averaging across reciprocals. Genes whose expression in hybrids deviated more than 1.25-fold from that of either parent were considered to have nonconserved inheritance and were classified in the following categories (Gibson *et al*. 2004; McManus *et al*. 2010): additive, if hybrid expression was greater than one parental and less than the other; dominant, if hybrid expression was similar to one of the two parents; and overdominant (underdominant), if hybrid expression was greater (less) than both parents.

**Fig. 4.**
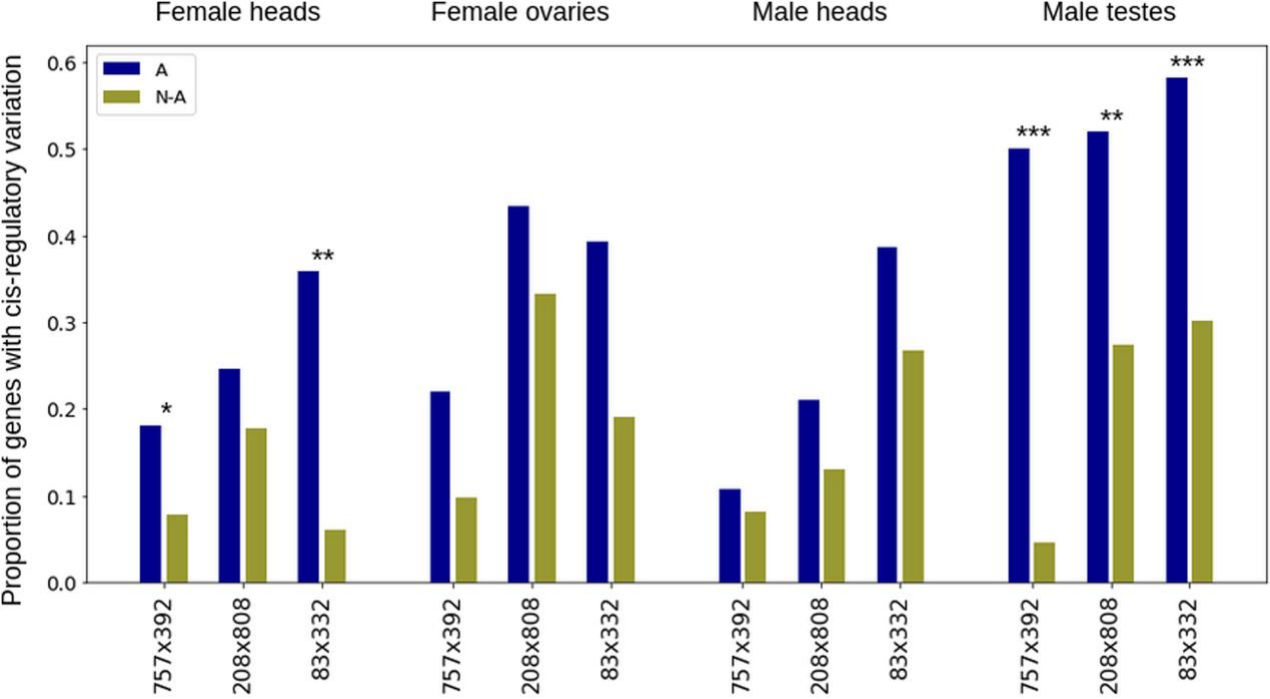
Proportion of additive and nonadditive genes with CR divergence. Blue and ocher bars display the proportion of genes showing CR variation of those additive (A) and nonadditive (N-A) genes for each sex, tissue, and cross. Stars indicate significance in the proportion of CR effects between A and N-A genes within samples: ***P-value > 0.001, **P-value < 0.01, *P-value < 0.05, and nonsignificant otherwise (2-proportions *z*-test).

Overall, we found that 20.5–41.2% of genes have additive effects, 50.6–65.1% have dominance effects in both directions, and 1.0–14.0% (1.0–18.8%) have underdominance (overdominance) effects (Supplementary Fig. 5). In agreement with previous results, we found that additive genes have more CR variation than nonadditive genes, consistent in all tissues, sexes, and crosses (Fig. 4), although this is only significant for a subset of these. However, we did not observe an enrichment for any inheritance pattern in any sex or tissue across different types of analyses (when analyzing both reciprocals together or separately or by using a statistical test rather than fold differences, Supplementary Figs. 5 to 7). In particular, we did not find an overall enrichment of additive effects in the testis (Supplementary Fig. 5), as might be expected from the observed enrichment in CR effects in this tissue. On the other hand, we did find that testes display the highest proportion of CR variation amongst additive genes (Fig. 4).

## Discussion

### Absence of PO and MG effects and inconsistent *trans*-regulatory effects between crosses and tissues

We estimated *cis* and *trans* effects acting on gene expression by comparing allelic expression between parental and hybrid lines in heads and gonads of *D. melanogaster* using three separate crosses between lines from the DGRP panel. Contrary to what has been found in other within-species studies of *cis* and *trans* regulation (Signor and Nuzhdin 2018), we find more *cis*- than *trans*-regulatory effects. This may simply be due to our choice of threshold to call 1 vs the other, which is necessarily arbitrary and may introduce biases. However, this should not be an issue here, since we apply the same approach to all samples and are primarily interested in comparing the different types of regulatory effects across sexes, tissues, and replicate crosses rather than providing direct quantifications of these effects. Since no clear differences between samples were found for *trans* effects and the method of Takada *et al*. (2017) applies linear modeling rather than simple cutoffs to infer *cis* effects, we focused on the results of the latter approach for the rest of the study. Their strategy detects CR together with PO and MG effects on gene expression. In agreement with previous results (Wittkopp *et al*. 2006; Coolon *et al*. 2012; Chen *et al*. 2015; Takada *et al*. 2017), we found no PO and minimal MG effects only in males (Supplementary Table 1), which suggests that the latter might reflect X-downstream effects. Overall, this confirms the fact that hybrids derived from reciprocal crosses have almost exactly the same patterns of expression in *D. melanogaster*, which is why we pooled them for the rest of the analyses.

### Testis-specific regulatory architecture of gene expression

Most of the sex-specific analyses of the regulatory architecture in *Drosophila* have been performed on whole bodies, which can mask the true extent of expression variation. Despite this, ample evidence was found for independent effects of *cis* variants on male and female gene expression even when the genes involved were sex-biased in expression (Gibson *et al*. 2004; Coolon *et al*. 2013, 2015; Meiklejohn *et al*. 2014). Here, we show that, at least for *cis* variants, these differences were most likely driven by testis-specific CR mechanisms of gene expression. Since we only sampled 1 somatic organ as a control (heads), it is possible that other sexually dimorphic tissues also display sex differences in allelic expression and that sex-specific CR interactions are a more general mechanism contributing to sex-specific expression and overall sexual dimorphism. However, this effect is likely to be strongest in the testis, since, in *Drosophila*, this tissue is known to have a different regulatory architecture compared to ovary and somatic tissues (Landeen *et al*. 2016; Witt *et al*. 2021). While in the ovary expression is primarily driven by a combination of transcription factors, the more broadly open chromatin of the testis contributes greatly to expression in this tissue (Witt *et al*. 2021). Polymorphic SNPs have been shown to lead to substantial changes in the chromatin state in various *Drosophila* tissues (Huynh *et al*. 2023), which potentially explains why we observe that *cis* variation in the testis is more abundant but also affects different genes than in other tissues. Surprisingly, given the limited role of transcription factors in driving expression in the testis, we did not find a systematic reduction in *trans* effects for this tissue compared to heads or ovaries. Instead, inconsistencies in *trans* effects were detected between crosses and tissues, highlighting the limited power to draw conclusions from just a single comparison. Similarly, no clear difference was detected for the inheritance patterns across the different tissues, a somewhat unexpected result given two observations. First, in crosses between *Drosophila mauritania* and *Drosophila simulans*, *cis* variants are more likely to have different effects in males and females (Meiklejohn *et al*. 2014). Second, additive changes are more likely to be controlled in *cis* (Lemos *et al*. 2008; McManus *et al*. 2010; Meiklejohn *et al*. 2014), a pattern which also holds for all of our crosses, such that an excess of additive variation might have been expected in testes. While this is not the case, we do find that the relative enrichment in CR effects in additive genes with respect to nonadditive genes is strongest in testes relative to the other tissues (Fig. 4).

### Selective pressures acting on sex-biased expression

Following Mishra *et al*. (2022), we divided genes according to their level of SB to try to gain new insights into what selective pressures are shaping gene expression with different levels of dimorphism. Analyses within one species are limited for this purpose, as both stronger stabilizing and directional selection on expression can lead to fewer polymorphic *cis* variants, while only the latter would lead to large numbers of fixed differences between more distant populations and species. Similarly, without a clear neutral control, more frequent detection of *cis* effects for one class of genes can be diagnostic of either balancing selection (e.g. due to intralocus sexual conflict overexpression levels) or decreased selective constraint. Despite these caveats and based on our knowledge of expression divergence for different tissues, some selective scenarios appear more likely. For instance, in heads, all but the most sex-biased genes are frequently under *cis* effects in both females and males. Given the rapid turnover of sex-biased genes in heads of *Drosophila* species (Khodursky *et al*. 2020), 1 possibility is that very sex-biased genes are under stronger directional selection. However, a recent study comparing within- and between-species divergence of *Drosophila* head expression found no evidence of positive selection acting on sex-biased genes and some support for balancing selection acting on the expression of female-biased genes (Khodursky *et al*. 2020). Furthermore, they found evidence of strong genetic correlations between male and female expression in this tissue (Khodursky *et al*. 2020), consistent with the near absence of sex-by-*cis* effects in our head data. Taken together, these patterns may suggest that sexual antagonism overexpression leads to the maintenance of more *cis* variants in unbiased and moderately biased genes, generally in line with the predictions of Mishra *et al*. (2022). Interestingly, in that study, no such effect was found, with female-biased genes showing more *cis* effects than other categories of sexual dimorphism. One difference may be the distance between parental lines used, since ours were derived from the same North American population, and theirs compared one North American and one South African. Over substantial periods of reproductive isolation, balancing selection due to sexual conflict will act to reduce divergence, and this may contribute to the difference observed here.

The patterns we obtain for gonads are more complex and differ when we estimate *cis* effects in ovaries vs testes, consistent with the prevalence of *cis*-by-sex effects in this tissue. In ovaries, strongly male-biased genes harbor weaker or fewer *cis* effects than other genes. In the testis, no clear pattern emerges, with different categories of sex-biased genes harboring the highest strength of *cis* effects in different crosses. Two hypotheses could explain why male-biased genes behave differently in the two sexes. First, testis-biased genes may be depleted of regulatory variants (leading to reduced *cis* effects in the ovary), with each variant having a disproportionately large effect on gene expression in the testis (restoring them in the testis). Second, testis-biased genes may have normal levels of diversity at regulatory sites, but these regulatory variants do not lead to detectable changes in expression in the ovary. While the approaches used here do not infer where *cis* variants are located relative to the genes they regulate, diversity data at 5′ UTRs in *D. melanogaster* and *D. simulans* do not support reduced diversity upstream of male-biased genes (if anything, there may be a slight excess of variants; Lawniczak *et al*. 2008; Campos *et al*. 2018). It therefore seems more likely that *cis* variants at testis-biased genes do not lead to changes in ovary expression. Whether this represents a true biological difference or a limitation of the method to detect *cis* effects when gene expression is low, as is the case of testis-biased genes in the ovary, is currently unclear. However, it is in line with the observation that many *cis*-eQTLs that modulate male expression in *D. melanogaster* do not do so in females even if they affect genes that are expressed in both sexes (Massouras *et al*. 2012). In any case, we find no substantial evidence of increased *cis* variation for genes at intermediate SB in the gonad, suggesting that sex differences in allelic usage in this organ may be sufficient to avoid widespread sexual conflict overexpression. Furthermore, the fact that many CR interactions shaping testis expression do not affect expression in ovaries may partly explain why male-biased gene expression is subject to few constraints and can therefore evolve fast, in line with what is often observed. More generally, these results highlight the need for approaches that incorporate the identification of *cis* variants with their cumulative regulatory effects on genes, as well as diversity and divergence estimates, in order to fully understand the mutation-to-*cis* effect relationship and the selective pressures acting on expression and their regulatory variants.

## Supplementary Material

jkad121_Supplementary_Data

## Data Availability

All RNA-seq reads have been deposited to the NCBI short read archive under BioProject number PRJNA945803. The tables of overall allelic expression in parentals and hybrids as counts and TPM estimated using Kallisto are in Supplementary Datasets 1 and 2, and the allele-specific parental and hybrid expression data estimated using ASE-Tigar are in Supplementary Datasets 3 and 4 as counts and FPKM, respectively. Supplementary Datasets are available at https://doi.org/10.15479/AT:ISTA:12933. Supplemental material available at G3 online.
