# Supplementary material for: Sex-specific estimation of cis and trans regulation of gene expression in heads and gonads of Drosophila melanogaster
Source: G3 (Bethesda). 2023 Jun 1;13(8):jkad121. doi: 10.1093/g3journal/jkad121 (PMC10411594; doi:10.1093/g3journal/jkad121)
Supplement: jkad121_Supplementary_Data [file jkad121_supplementary_data.pdf]

Dataset S1. Overall count data in parentals and hybrids estimated using Kallisto. Each column contains count gene expression for a particular sample. Each sample is labeled as follows: 1 2 3. 1 indicates the maternal (F) and paternal (M) lines of the hybrid cross. 2 is the tissue and sex: fh (female heads), fo (female ovaries), mh (male heads) and mt (male testes). 3 is the replicate: R1 or R2. Expression data for samples 392F x 757M mh R2, 392M x 392F mt R2 and 808M x 208F mt R2 are removed due to low quality, and not used in any analysis. The last two columns (chr and start) are the chromosome and position of the gene.

The dataset corresponds to Dataset S1 in this link: https://doi.org/10.15479/AT:ISTA:12933

Dataset S2. Overall TPM data in parentals and hybrids estimated using Kallisto. Each column contains TPM gene expression for a particular sample. Each sample is labeled as follows: 1 2 3. 1 indicates the maternal (F) and paternal (M) lines of the hybrid cross. 2 is the tissue and sex: fh (female heads), fo (female ovaries), mh (male heads) and mt (male testes). 3 is the replicate: R1 or R2. Expression data for samples 392F x 757M mh R2, 392M x 392F mt R2 and 808M x 208F mt R2 are removed due to low quality, and not used in any analysis. The last two columns (chr and start) are the chromosome and position of the gene. This dataset is used for the inheritance patterns classification.

The dataset corresponds to Dataset S2 in this link: https://doi.org/10.15479/AT:ISTA:12933

Dataset S3. Overall parental and allele-specific hybrid count expression estimated using ASETigar**.** Each column contains count gene expression for a particular sample. Parental overall expression data has been estimated using the ASETigar pipeline (see Methods) so that the estimates are comparable to the allelic expression in the hybrids. Expression for each specific pairwise comparison is labeled as 1_2_34_5. 1 and 2 are the two lines being compared, 3 is the tissue and sex: fh (female heads), fo (female ovaries), mh (male heads) and mt (male testes), 4 is the replicate: R1 or R2. 5 is the line (corresponding with 1 or 2) for which expression is estimated. Hybrid allele-specific expression estimates are labeled as 1Fx2M_3_4_5. 1 and 2 indicate the maternal and paternal lines of the hybrid cross, respectively. 3 is the tissue and sex, 4 the replicate and 5 the line (corresponding with 1 or 2) for which allele-specific expression is estimated. Expression data for samples 392F x 392M mt R2, 208Fx808M_mt_R2 and 392Fx757M_mh_R2 are removed due to low quality and not used in the analysis. The last two columns (chr and start) are the chromosome and position of the gene. This dataset is used to estimate cis regulatory effects via the two

described methods: only hybrid allele-specific expression was used for CR, PO and MG estimates following Takada *et al.* (2017)'s pipeline; parental and hybrid allelic expression was used for the cis vs trans estimates of regulatory variation following McManus *et al.* (2010)'s approach.

The dataset corresponds to Dataset S3 in this link: https://doi.org/10.15479/AT:ISTA:12933

Dataset S4. Overall parental and allele-specific hybrid FPKM expression estimated using ASETigar**.** Each column contains FPKM gene expression for a particular sample. Parental overall expression data has been estimated using the ASETigar pipeline (see Methods) so that the estimates are comparable to the allelic expression in the hybrids. Expression for each specific pairwise comparison is labeled as 1_2_34_5. 1 and 2 are the two lines being compared, 3 is the tissue and sex: fh (female heads), fo (female ovaries), mh (male heads) and mt (male testes), 4 is the replicate: R1 or R2. 5 is the line (corresponding with 1 or 2) for which expression is estimated. Hybrid allele-specific expression estimates are labeled as 1Fx2M_3_4_5. 1 and 2 indicate the maternal and paternal lines of the hybrid cross, respectively. 3 is the tissue and sex, 4 the replicate and 5 the line (corresponding with 1 or 2) for which allele-specific expression is estimated. Expression data for samples 392F x 392M mt R2, 208Fx808M_mt_R2 and 392Fx757M_mh_R2 are removed due to low quality and not used in the analysis. The last two columns (chr and start) are the chromosome and position of the gene.

The dataset corresponds to Dataset S4 in this link: https://doi.org/10.15479/AT:ISTA:12933

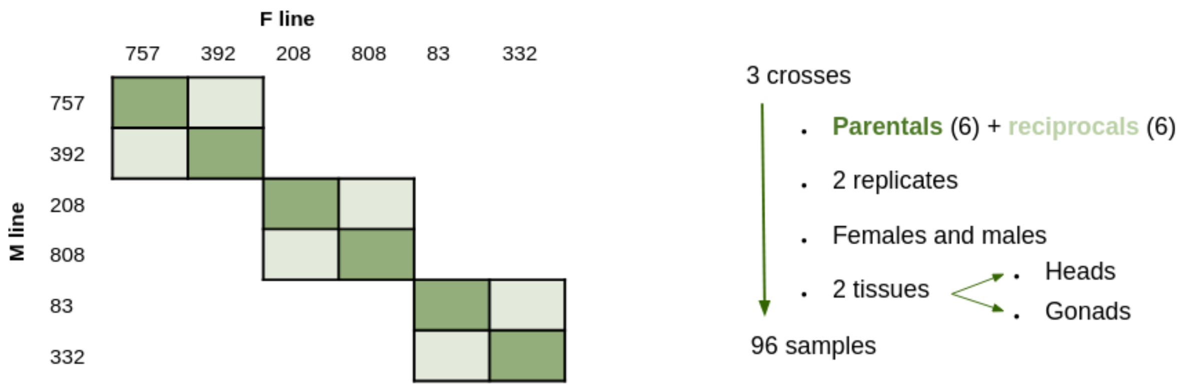

Figure S1. Outline of the experimental design. We randomly selected DGRP 6 lines without *Wolbachia* infection and main inversions and matched them into three pairs: DGRP-757 x DGRP-392, DGRP-208 x DGRP-808 and DGRP-83 x DGRP-332. For each pair, we performed within- and both reciprocal between-line crosses and obtained sex-specific head and gonad gene expression for two replicates of each sample.

Female heads						
	757x392		208x808		83x332	
N genes	5897	1096	5854	1047	5812	1117
CR	610	87	460	106	447	68
PO	15	4	10	4	4	0
MG	16	1	7	0	3	0

Male heads						
	757x392		208x808		83x332	
N genes	5767	1092	6135	1118	6291	1166
CR	484	75	476	78	487	69
PO	23	576	0	641	2	602
MG	139	55	106	68	148	61

Female ovaries						
	757x392		208x808		83x332	
N genes	5124	1049	4783	985	4621	992
CR	400	74	392	73	580	85
PO	0	6	0	1	10	2
MG	27	1	84	4	40	7

Male testes						
	757x392		208x808		83x332	
N genes	6724	1182	6576	1138	6898	1217
CR	1110	74	918	69	1076	60
PO	30	667	25	612	7	713
MG	26	65	295	47	81	44

Autosomes X chromosome

Table S1. Number of genes showing significant cis-regulatory (CR), parent-of-origin (PO) and maternal genotype (MG) effects in each tissue, sex and cross for both autosomal (grey) and X-linked (white) genes.

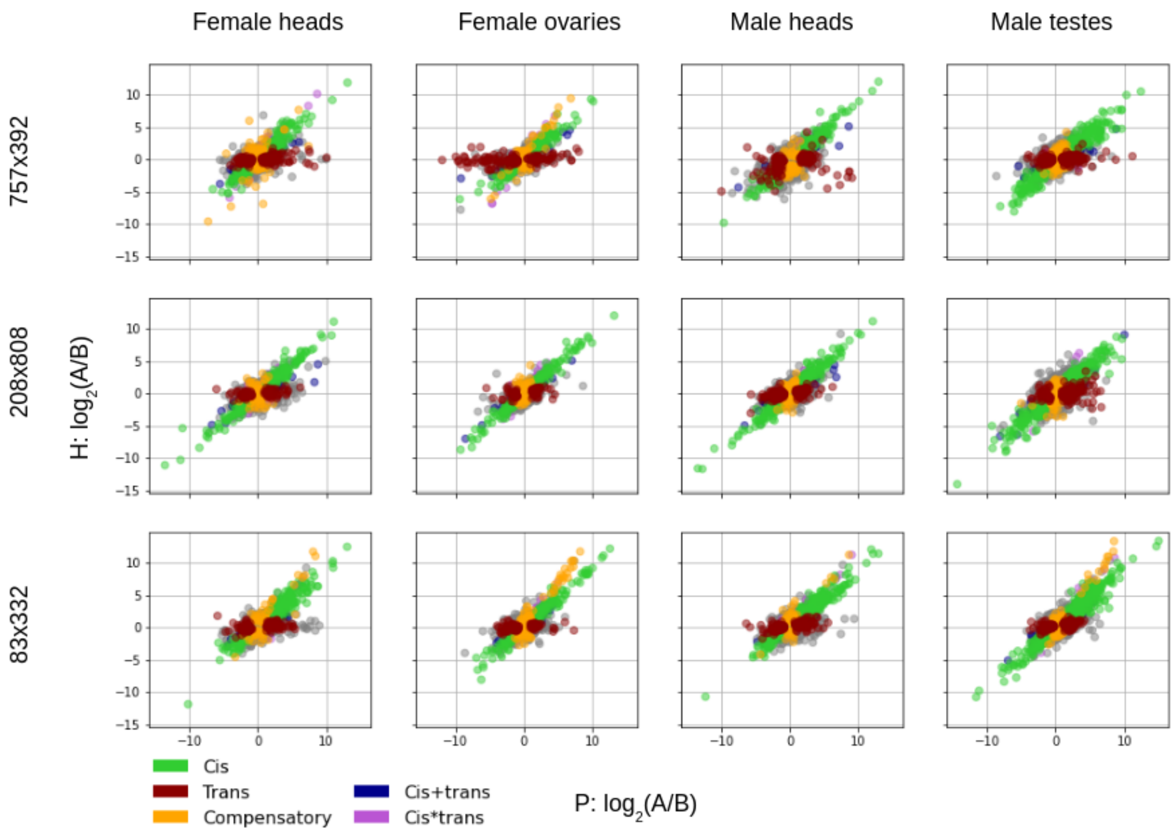

Figure S2. Inferred cis and trans regulatory mechanisms. Scatter plots of the relative averaged allele-specific expression levels in parental (P) vs hybrid (H, averaged across reciprocals) datasets in each sex, tissue and cross. Each dot is a gene and is color-coded according to the inferred mechanism of expression regulation: cis (green), trans (red), compensatory (yellow), cis+trans (blue) and cis*trans (purple).

A

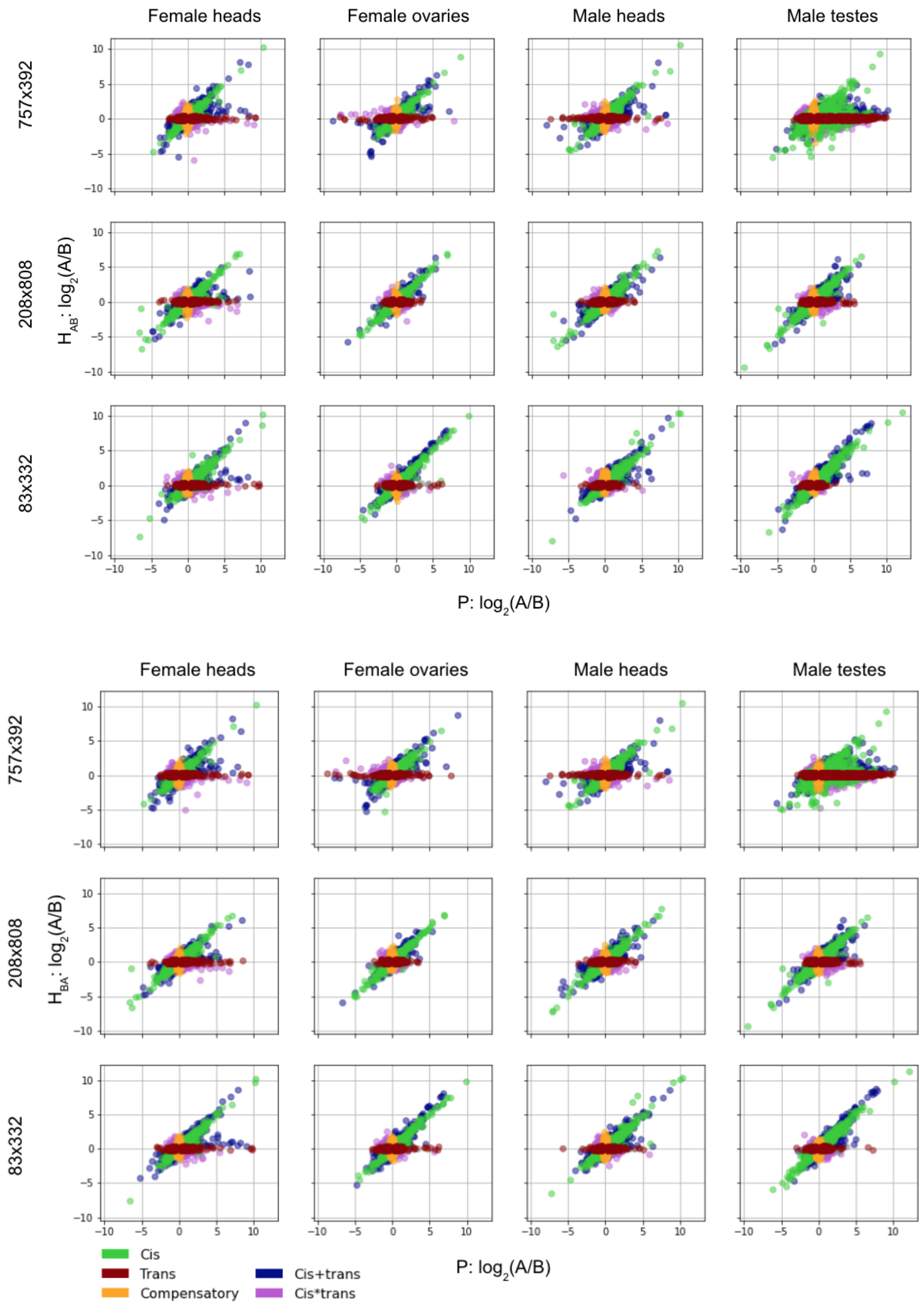
Female heads
Female ovaries
Male heads
Male testes
757x392
208x808
83x332
H_{AB}: $\log_2$(A/B)
H_{BA}: $\log_2$(A/B)
P: $\log_2$(A/B)
Cis
Trans
Compensatory
Cis+trans
Cis*trans

B

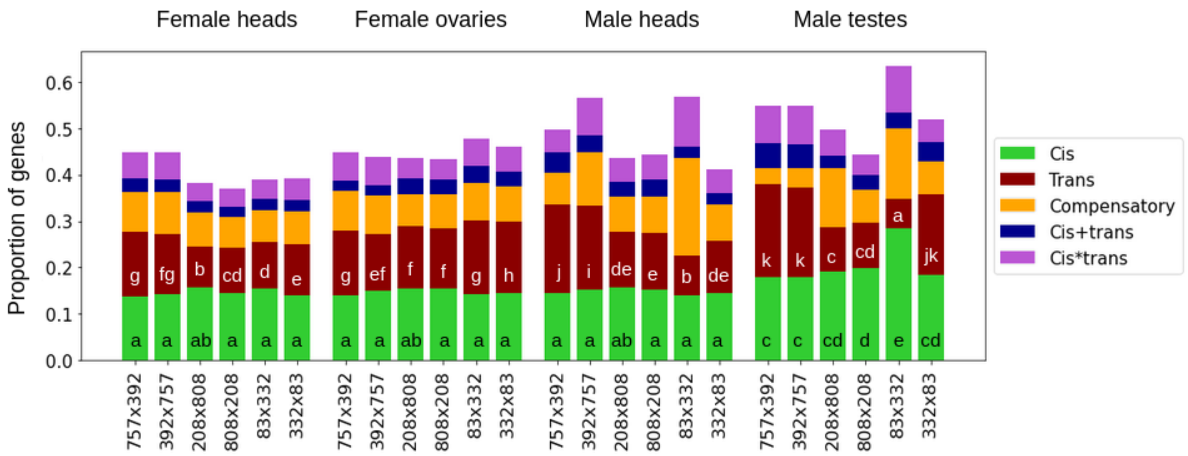

Figure S3. Inferred cis and trans regulatory mechanisms in both reciprocal crosses separately. A) Scatter plots of the relative allele-specific expression levels in parentals (P) vs hybrids (H) in each sex, tissue and reciprocal (AxB and BxA in first and second panels, respectively) for each cross. Each dot is a gene and is color-coded according to the inferred mechanism of expression regulation: cis (green), trans (red), compensatory (yellow), cis+trans (blue) and cis*trans (purple). **B)** Proportion of genes with each inferred regulatory mechanism in each sex, tissue and reciprocal cross. Significance groups revealing differences in the proportion of genes classified as having cis (black) and trans (white) regulation across all samples (two-proportions z-test at p-value <0.05) are denoted by different letters (a–e and a-k).

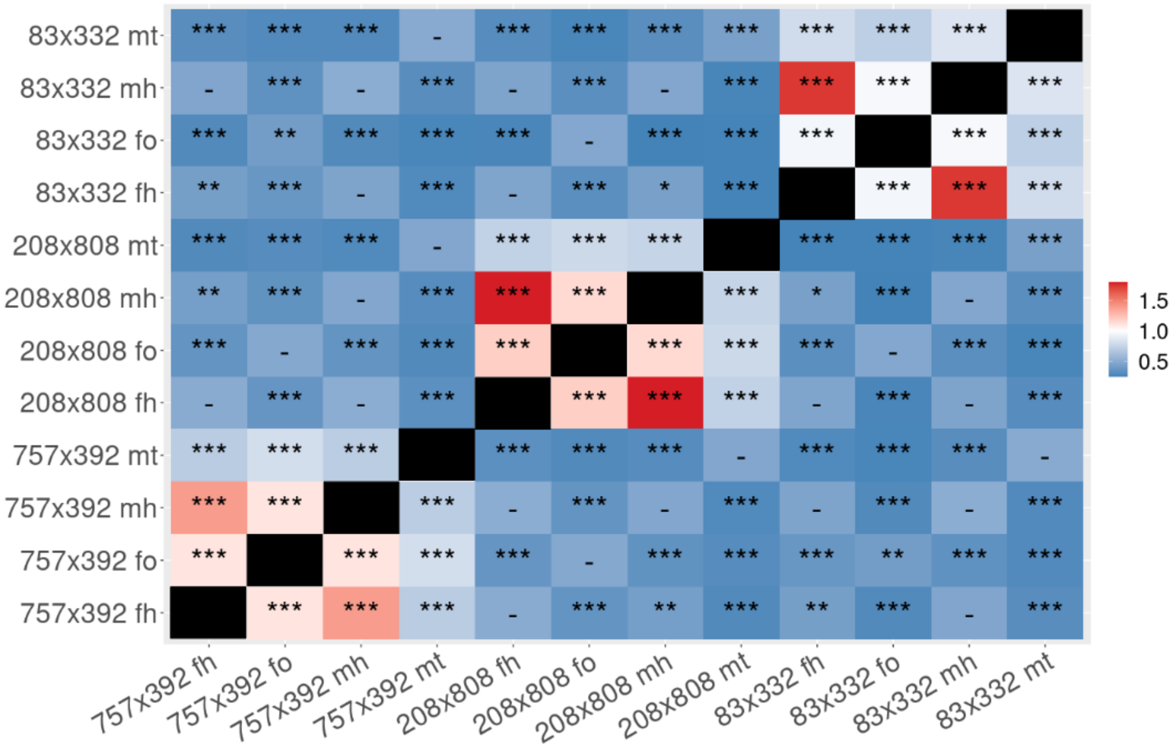

Figure S4. Overlap in cis-regulatory effects between samples. The color indicates the ratio between observed and expected overlap in CR effects between pairs of samples, red (blue) indicating larger (smaller) overlap than expected. fh: female heads, fo: female ovaries, mh: male heads, mt: male testes. The stars indicate significance: ***, p-value>0.001; **, p-value<0.01; *, p-value<0.05; non-significant (-), otherwise (Chi-squared tests).

A

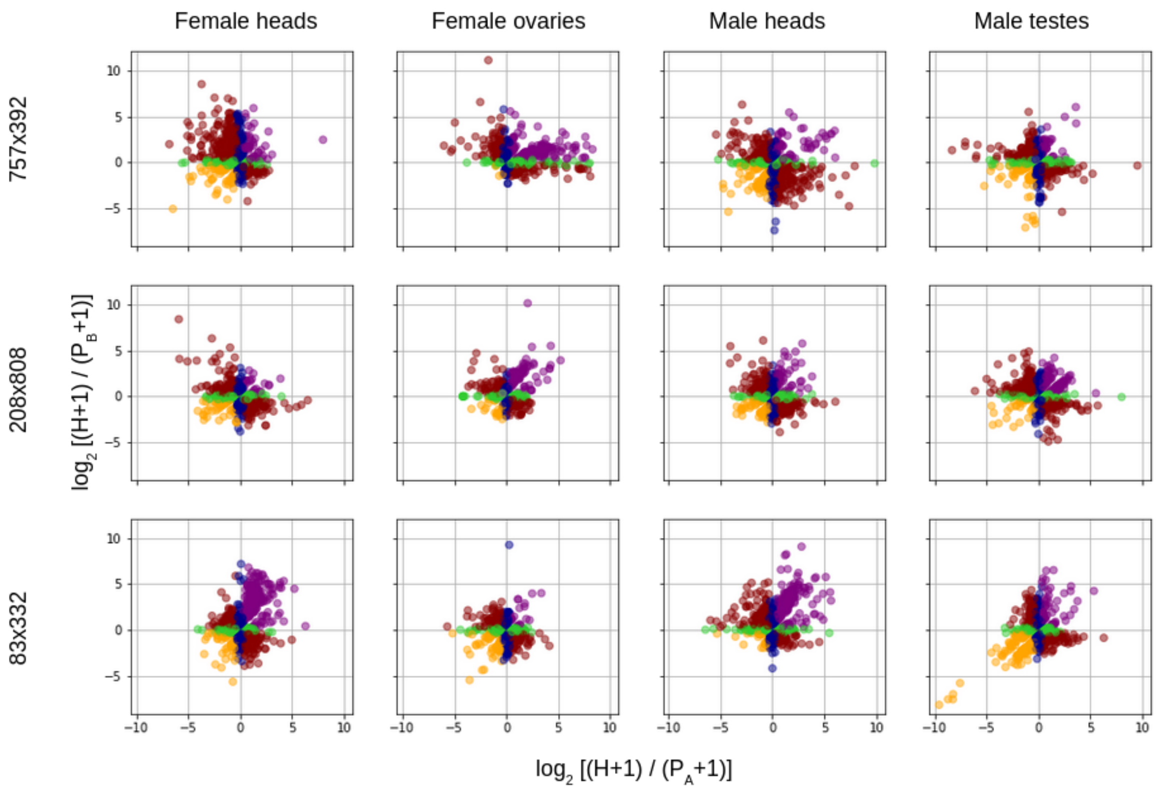

B

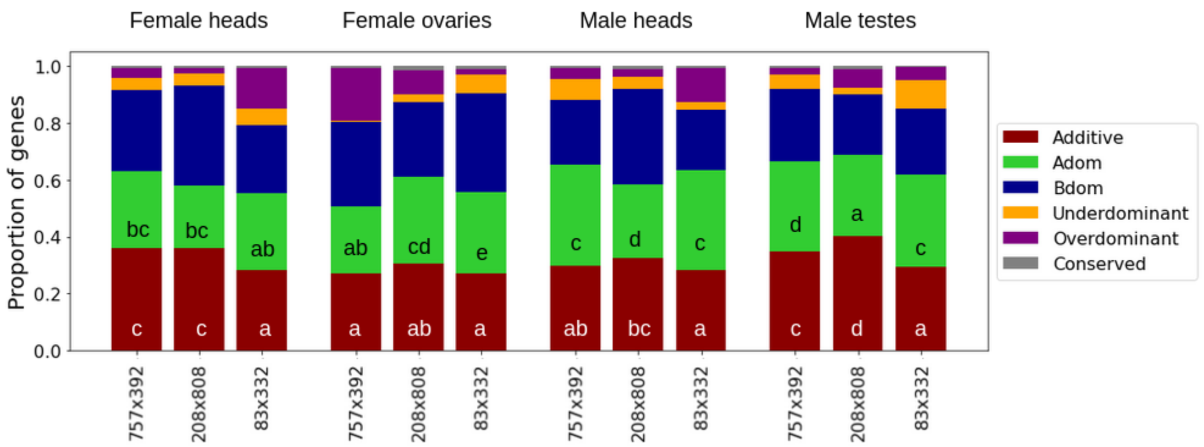

Figure S5. Inheritance patterns inferred using fold-differences. A) Scatter plots of the relative overall expression between hybrids (H) and parentals (P) in each sex, tissue and cross, averaged across reciprocals. Each dot is a gene and is color-coded according to the inheritance pattern, inferred using hierarchical classification based on fold expression differences between P and H. **B)** Proportion of genes with each inferred inheritance mechanism per sample. Significance groups revealing differences in the proportion of genes displaying additive and dominant –in both directions combined (in white and black respectively)– across samples (two-proportions z-test at p-value <0.05) are denoted by different letters (a–i and a-e).

A

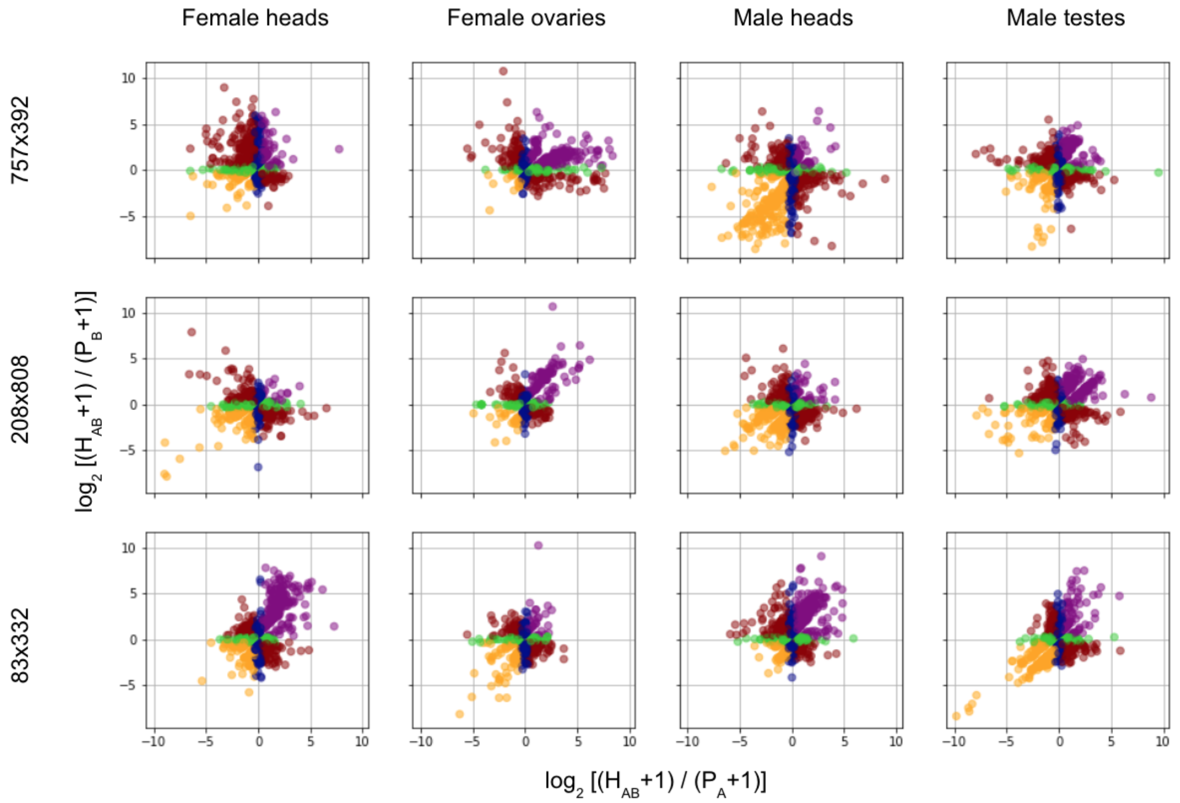
Female heads
Female ovaries
Male heads
Male testes
757x392
208x808
83x332
log2 [(HAB+1) / (PB+1)]
log2 [(HAB+1) / (PA+1)]

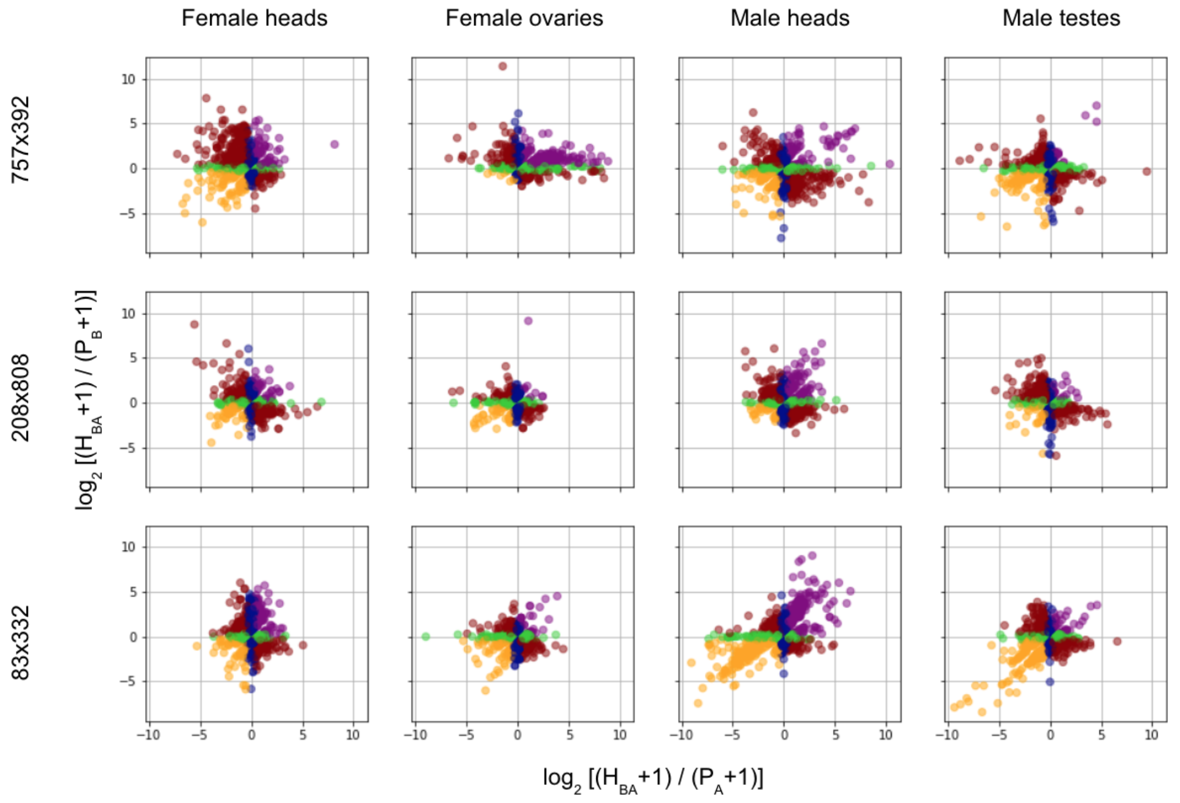
Female heads
Female ovaries
Male heads
Male testes
757x392
208x808
83x332
log2 [(HBA+1) / (PB+1)]
log2 [(HBA+1) / (PA+1)]

B

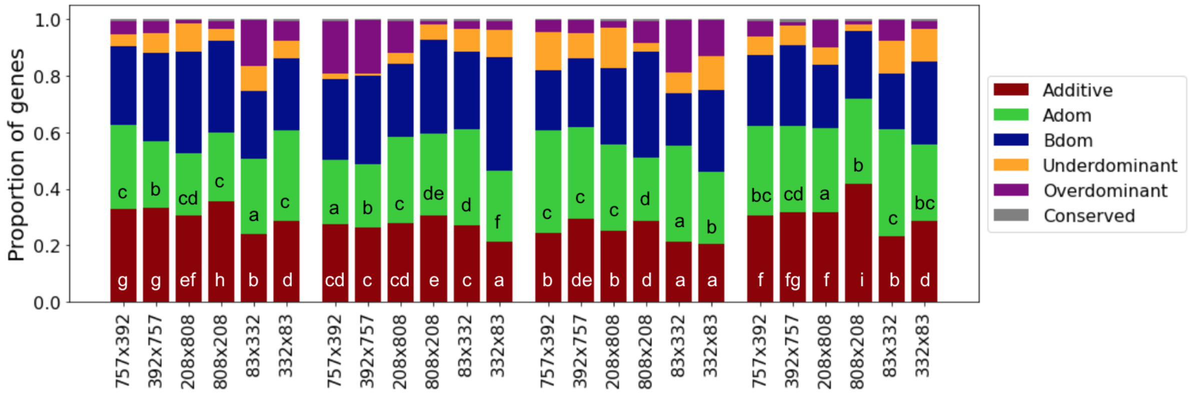

Figure S6. Inheritance patterns inferred using fold-differences in reciprocal crosses separately. A) Scatter plots of the relative overall expression between hybrids (H) and parentals (P) in each sex, tissue and both reciprocals (AxB and BxA in first and second panels, respectively) for each cross. Each dot is a gene and is color-coded according to the inheritance pattern, inferred using hierarchical classification based on fold expression differences between P and H. **B)** Proportion of genes with each inferred inheritance mechanism per sample. Significance groups revealing differences in the proportion of genes displaying additive and dominant –in both directions combined– (in white and black respectively) across samples (two-proportions z-test at p-value <0.05) are denoted by different letters (a–i and a-e).

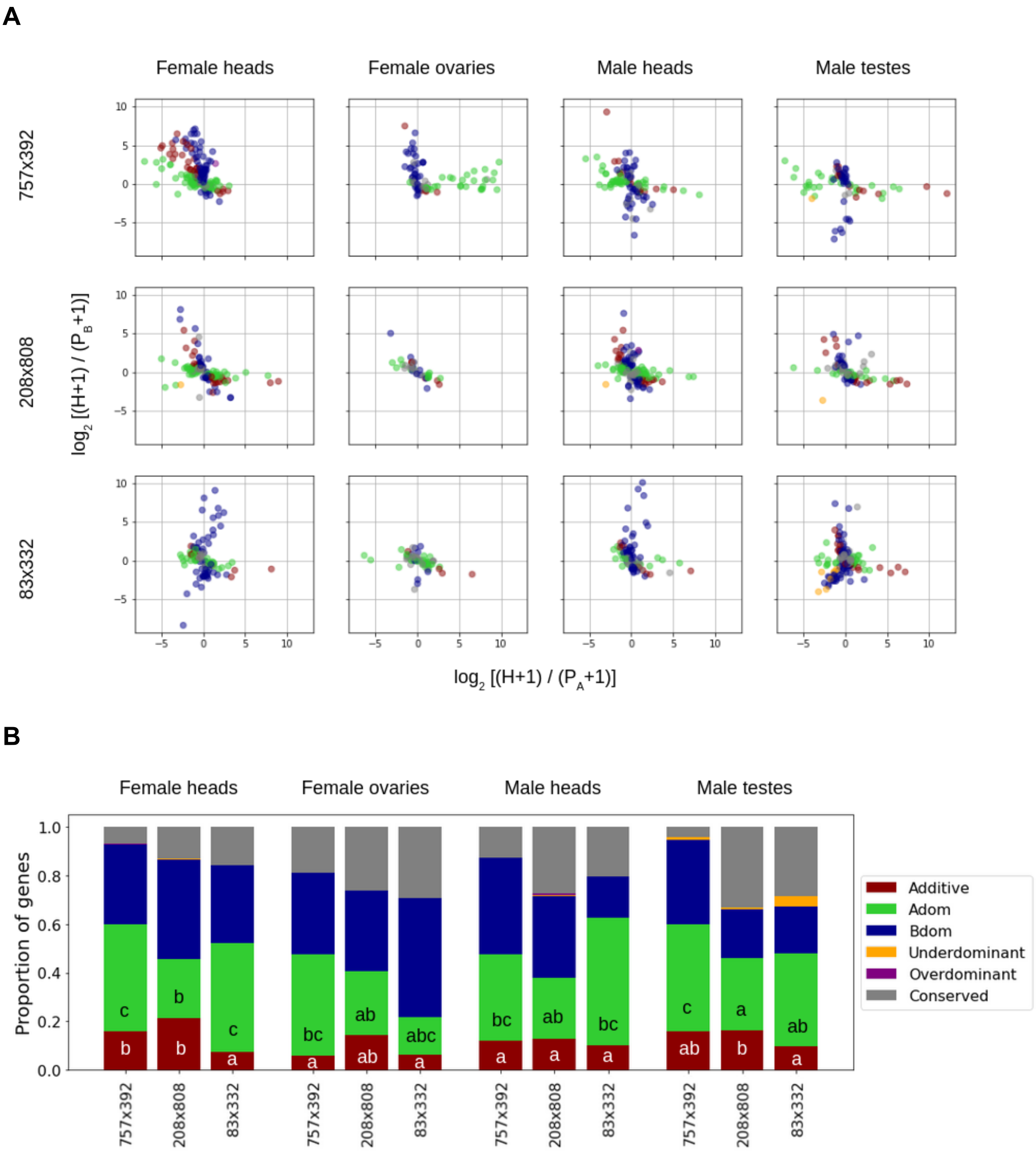

Figure S7. Inheritance patterns inferred using statistical tests. Instead of fold differences, the classification into the various inheritance patterns was done via statistical testing for differences in expression between parentals and hybrid crosses (pooling the reciprocals together) using DEseq2 at FDR<0.05. **A)** Scatter plots of the relative overall expression between hybrids (H) and parentals (P) in each sex, tissue and cross. Each dot is a gene and is color-coded according to the inferred inheritance pattern, according to the legend in B). **B)** Proportion of genes with each inferred inheritance mechanism per sample. Significance groups revealing differences in the proportion of genes displaying additive and dominant –in both directions combined– (in white and black respectively) across samples (two-proportions z-test at p-value <0.05) are denoted by different letters (a–b and a-c).
